# Absolute quantification of *E*. *coli* virulence and housekeeping genes to determine pathogen loads in enumerated environmental samples

**DOI:** 10.1371/journal.pone.0260082

**Published:** 2021-11-29

**Authors:** K. B. Hoorzook, T. G. Barnard

**Affiliations:** Water and Health Research Centre, Faculty of Health Sciences, University of Johannesburg, Johannesburg, South Africa; University of Helsinki: Helsingin Yliopisto, FINLAND

## Abstract

Quantifying pathogenic genes with q-PCR in complex samples to determine the pathogen loads is influenced by a wide range of factors, including choice of extraction method, standard curve, and the decision to use relative versus absolute quantification of the genes. The aim was to investigate the standardisation of q-PCR methods to determine enumerated *E*. *coli* gene ratios grown with the IDEXX Colilert^®^ Quanti-Trays^®^ using enteropathogenic *E*. *coli* as the model pathogen. q-PCR targeting the *eaeA* and *gadAB* genes was used to calculate the *eaeA*: *gadAB* ratios for clinical strains collected between [2005–2006 (n = 55)] and [2008–2009 (n = 19)] using the LinRegPCR software and Corbett Research Thermal cycler software. Both programs grouped the isolates into two distinct groups based on the gene ratios although the Corbett Research Thermal cycler software gave results one log higher than the LinRegPCR program. Although the *eaeA*: *gadAB* ratio range was determined using extracted *E*. *coli* DNA, the impact of free DNA and other bacteria present in the sample needed to be understood. Standard curve variations using serially diluted extracted *E*. *coli* DNA, serially diluted pure *E*. *coli* culture followed by DNA extraction from each dilution with or without other bacteria was tested using the *eaeA* q-PCR to quantify the genes. Comparison of the standard curves showed no significant difference between standard curves prepared with diluted DNA or with cells diluted before the DNA is extracted (P = 0.435). Significant differences were observed when background DNA was included in the diluent or Coliform cells added to the diluent to dilute cells before the DNA is extracted (P < 0.001). The “carrier” DNA and Coliform cells enhanced the DNA extraction results resulting in better PCR efficiency. This will have an influence on the quantification of gene ratios and pathogen load in samples containing lower numbers of *E*. *coli*.

## Introduction

*Escherichia coli* (*E*. *coli*) is used as an indicator of faecal pollution in the water sector indicating the presence of other pathogenic bacteria [1]. *E*. *coli* strains can however be non-pathogenic (commensal) or pathogenic and capable of causing diseases ranging from diarrhoea, urinary tract infection (UTI), sepsis, gastrointestinal tract infections, hemolytic-uremic syndrome (HUS), to meningitis and inflammation of the meninges [2]. To understand the true risk and potential impact of these pathogenic *E*. *coli* it is important to determine the proportion of the *E*. *coli* community that is pathogenic, especially when considering the infectious dose ranges of the different pathogen *E*. *coli* strains [3]. This type of *E*. *coli* pathogen/commensal ratios are not typically known for environmental samples and could be useful in quantitative microbial risk assessments [4].

The IDEXX Colilert^®^ Quanti-Tray^®^ system, a standardised miniaturised most probable number (MPN) method has gained popularity for water analysis and has even been used to study *E*. *coli* levels in water samples. Omar et al. [5] developed a method for the study of pathogenic *E*. *coli* extracted from the Colilert^®^ Quanti-Tray^®^ using a multiplex PCR and showed that this could be used to study the *E*. *coli* population in a sample. The method was tested on, water (spring, borehole, tap, river, stream, domestic storage container, raw sewage, and final effluent), soil, stool, and toilet seat swab samples [5] and even dishcloths [6]. Membrane filtration with chromogenic Coliform/ *E*. *coli* media is generally used for enumerating *E*. *coli* from water samples. These methods are Environment Protection Agency (EPA) approved, certified, and accepted for *E*. *coli* analysis [7, 8]. However, these methods only highlight the presence of viable *E*. *coli* and cannot differentiate between the commensal *E*. *coli* and DEC strains. Further steps are required to identify specific *E*. *coli* strains, and these include biochemical tests, immunological assays, and molecular-based techniques such as polymerase chain reaction, which leads to increased cost and time.

Quantitative real-time PCR (q-PCR) have been used to detect diarrheagenic *E*. *coli* (DEC) types by targeting specific virulence genes [9] using either fluorescent dyes (SYBR^®^ Green) that bind to double-stranded DNA or fluorescent labelled sequence-specific probes such as Förster resonance energy transfer (FRET) probes; Molecular beacons (PNA based, light probes); Scorpions (duplex); Locked Nucleic Acid (LNA) probes and TaqMan^®^ probes [10–12].

q-PCR data are quantified either absolutely or relatively. Relative quantification that is generally used for most physiological and pathological studies, relies on the comparison between the expression of a target gene versus the expression of reference genes and the expression of the same set of genes in target samples versus a control sample. Relative quantification does not require a calibration curve or standards with known concentrations and the reference can be any transcript if its sequence is known [13–16]. Absolute quantification is widely used in microbial community analysis, allowing the quantification of the number of target genes in a community sample. Absolute quantification utilises the standard curve method to quantitate unknowns based on a known quantity. First, you create a standard curve; then you compare unknowns to the standard curve and extrapolate a value. It relies on an internal or external calibration curve, and these standard curves are used to derive the input template copy number and to ensure that the exact transcript copy number is determined [15, 17]. Absolute quantitative methods have been found to be more sensitive to gene expression variations caused by factors such as developmental and environmental variation [18, 19]. In this study, absolute quantification was used to develop standard curves for quantitative analysis.

There are problems associated with q-PCR analysis, where different sequences are often amplified with different amplification efficiencies, causing under/overestimation of input template copy numbers [20]. To overcome this is by diluting the input nucleic acid five to ten times with water. The quantification cycle (C_q_) values are plotted against the log of the known starting concentration value and from the slope of the regression line the amplification efficiency (*E*) is estimated. This method gives one *E* value for all dilution concentrations of the respective sequence. The *E* varies as the input concentration varies. When comparing to an unknown sample, the C_q_ value of the unknown sample is compared with the standard curve to determine the number of target copies in the unknown sample [21]. These analyses are performed on software that comes with the PCR system. The mainstream of qPCR data analysis is based on the direct application of the basic equation for PCR amplification *N*_*C*_ = *N*_*o*_ × *E*^*c*^ [22]. This basic equation for PCR kinetics states that the number of target copies after c cycles (Nc) is the starting number of targets (N0) times the PCR efficiency (shown as E) to the power c [23]. According to Ruijter et al. [11], various methods exist to assess curve analyses. Authors reported the similarities between these methods are striking as they are all based on the basic kinetics equation, and all calculate a target quantity using an efficiency value and a Cq value. A real difference in approach lies between those ‘constant efficiency’ algorithms and the methods that are based on continuously decreasing efficiency values [11]. In this study, the LinRegPCR program was selected to compare with the PCR system program. LinRegPCR uses a baseline estimation that is aimed at reconstructing an exponential phase in which the data points are on a straight line, the PCR efficiencies derived from these data points are less variable [11, 23].

An important factor that can also affect q-PCR which needs to be considered is the influence of background DNA in the DNA extraction process. Importantly to take note of the inconsistent way of preparing standard curves i.e., there is no standard method in creating standard curves. The MIQE guidelines do address the fact that there is a lack in the way q-PCR experiments are presented and interpreted, and that this should be addressed in promoting consistency and integrity of scientific research [14].

The aim of this manuscript was to investigate the standardisation of q-PCR methods to determine enumerated *E*. *coli* gene ratios grown with the IDEXX Colilert^®^ Quanti-Trays^®^ using enteropathogenic *E*. *coli* as the model pathogen. This includes absolute standard curve preparation, revealing the influence of the background DNA extraction process affecting quantitative analysis and how to calculate ratios between commensal and pathogenic *E*. *coli*. Therefore, creating a steppingstone in determining the presence of *E*. *coli* pathogen virulence genes, relative to its reference genes, for each *E*. *coli* pathotype in environmental samples.

## Methodology

The standard curve variations and the influence of the background DNA extraction process affecting quantitative analysis are highlighted within four standard curves. The methodology in developing these standard curves are as follows:

### Growth and maintenance of bacterial strains

Entero-haemorrhagic *Escherichia coli* (EHEC) (ESCCO 21), were cultured on Plate Count Agar (PCA) (Oxoid, UK) and incubated under aerobic conditions at 37°C for 16 hours. Clinical *E*. *coli* strains obtained between 2005–2006 (n = 55; Heine, 2007) and 2008–2009 (n = 19; Ampath Laboratories) were cultured onto *E*. *coli* Coliform Chromogenic Media (Oxoid, UK) and incubated under aerobic conditions at 37°C for 16 hours. Single colonies that appeared purple on the selective *E*. *coli* media were enriched in Nutrient broth (Oxoid, UK) and incubated under aerobic conditions at 37°C for 16 hours with rotation at 200 rpm.

### DNA extraction

DNA was extracted as described by Omar et al. [5] from 2 ml of the overnight cultures grown for each bacterial isolate adjusted to an optical density (OD_600nm_) of 1.0. The extracted DNA was used as a template for the q-PCR reactions. The sample for DNA extraction was also collected from Colilert^®^ Quanti-Trays^®^ wells containing Coliform growth from environmental water samples as described by Omar et al. [5].

### Quantitative real-time polymerase chain reaction (q-PCR)

All q-PCR reactions were performed in a Corbett Research Thermal cycler (now Rotor-Gene^®^) (Qiagen^®^) in a total volume of 20 μl. Each reaction consisted of 1 X 2 μl Qiagen^®^ PCR buffer mix; 0.1 μl Hotstart Taq^®^ DNA polymerase and 0.6 μl dNTP mix; 1 μl of a 3 μM *eaeA* TaqMan^®^ (r-AGT CGA ATC CTG GTG CGG C-q) or *gadAB* LNA probe (r-CGG TGR CMG GAM GCR A-q); 2 μl of Mg^2+^; 1 μl of each 5 mM *eaeA* forward primer (5’-TGT TGC TTT GTT TAA TTC YGA TAA GC-3’) and reverse primer (5’-GGA ATC GGA GTA TAG TTT ACA CCA A-3’), or 1 μl of each 5 mM *gadAB* forward primer (5’-GCG GAA GTC CCA GAC GAT ATC C-3’) and reverse primer (5’-GCT ACA CGT ACA GCT ACA GCT A-3’); 3 μl of sample DNA and 9.3 μl PCR grade water. Diluted 10^−1^ and 10^−2^ DNA of referenced EHEC or commensal *E*. *coli* were included with all q-PCR reactions.

The PCR reactions were subjected to a 2-step RT-PCR protocol, an initial activation step at 95°C for 15 min, after heating, DNA was amplified for 35 cycles at 94°C hold for 15 sec and 55°C hold for 60 sec. Thereafter, absolute quantification was performed to determine the exact number of *eaeA* copies present in the sample by relating the PCR signal to the optimised standard curve.

### Standard curve construction using isolated EHEC DNA

Using the optimized q-PCR protocols for EHEC/EPEC (*eaeA*) and *E*. *coli* acid tolerance gene (*gadAB*) probe, standard curves were created by diluting the extracted EHEC DNA tenfold in PCR grade water in triplicate and each q-PCR was performed in duplicate for each gene. The extracted DNA was quantified in ng/μℓ using the Qubit^TM^ fluorometer (Invitrogen USA). This was converted into copies/μℓ using Eq 1 below [24, 25] and used as the starting template concentration “Table 1” in the q-PCR analysis.

**Table 1:** Starting template concentration added for the standard curves.

Pathogen	Probes	Initial concentration (g/ℓ)	Copies/3μℓ
EHEC/EPEC	*eaeA*	0.00147	8.7 x10^5^
Commensal	*gadAB*	0.00493	2.9 x10^6^


$$Number\;of\;initial\;copies/\mu l=\frac{DNA\;concentration\times Avogadro^{\prime }s\;constant}{Genome\;size\times Avergae\;weight\;of\;basepair}$$ (Eq 1)

Where the DNA concentration is given in g/L, Avogadro’s constant is 6.022 x10^23^mol^-1^, the size of the complete *E*. *coli* genome is 4.7 x 10^6^ bp is and 660 gxmol^-1^ is used as the average weight of the base pair.

### Gene ratios of clinical isolates

DNA was extracted from the 74 *E*. *coli* isolates and the extracted DNA was used to measure the *eaeA* and *gadAB* gene copy numbers using the standard curves constructed with the EHEC DNA. The *eaeA* and *gadAB* standard curves were imported to measure copies for the unknown strains to obtain a ratio between *eaeA*: *gadAB* using the Corbett Research Thermal cycler machine. The q-PCR results of the unknown strains were measured by the Corbett Research Thermal cycler machine software, the raw data from the Corbett Research Thermal cycler machine software was thereafter imported into the LinRegPCR analysis program [22] to compare the q-PCR results between the two programs.

### Influence of DNA and other bacteria on EHEC standard curves

**Fig 1:**
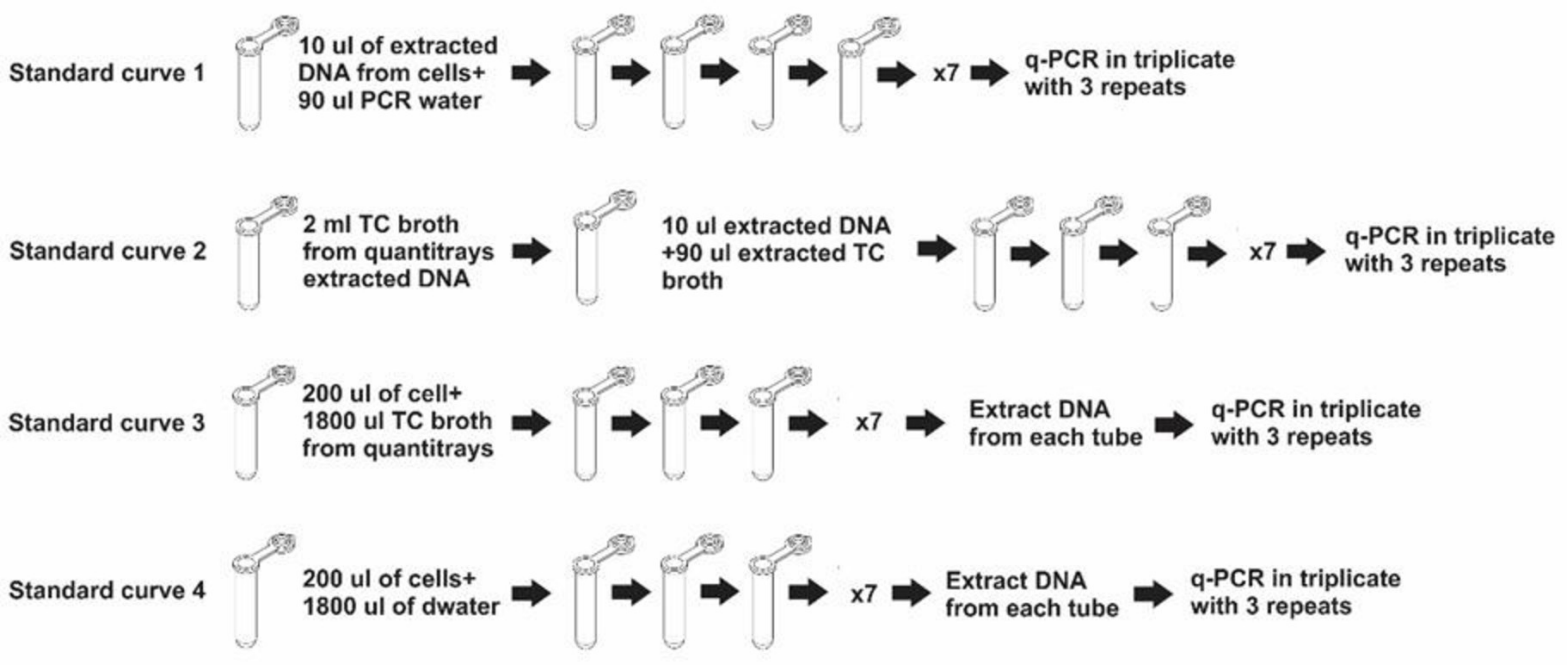
Schematic representation of how the standard curves were constructed.

Absolute quantification utilizes a standard curve in which extracted DNA is diluted tenfold in water “Fig 1 Standard curve 1”. An additional three standard curves preparations were included in this study. These standard curve variations were used to measure the *eaeA* gene as a representative of the other genes detected for *E*. *coli*.

“Standard curve 2 Fig 1” was created by diluting extracted EHEC DNA tenfold in extracted coliform DNA extracted from the Colilert^®^ Quanti-Trays^®^ wells and was used as background DNA. “Standard curve 3” was created by diluting EHEC cells tenfold in constant volume and concentration of broth containing the coliforms from the Colilert^®^ Quanti-Trays^®^. DNA was extracted from each dilution as described above then used in q-PCR analysis. “Standard curve 4” was created by diluting EHEC cells tenfold in sterile distilled water. Thereafter, DNA was extracted from individual dilution tubes and used for the q-PCR. In all cases, the dilutions and extractions were done in triplicate for each of the standard curves and each dilution was analysed in duplicate with q-PCR.

Data was used to draw standard curves and the PCR efficiency was estimated through the linear regression of the dilution standard curves. Statistical analysis was performed using the coefficient of determination (R^2^) calculated from the linear regression to compare the 4 standard curves using the assumption that the higher the R^2^ value or closest to 1 indicates a more robust model [26].

### Calculations and Statistical analysis

#### Calculations used for q-PCR

All programs plot a standard curve using designated wells or Microsoft Excel^®^ to draw xy plots with the log template amount as the x-value and the threshold cycle (C_q_) as the y-value. Eq 2 calculates a line representing the best fit for the standard curve using the least square method of linear regression [27].


y=mx+b (Eq 2)

Where y is the Quantification cycle (C_q_), m is the slope, x is the log_10_ template input and b is the y-intercept.

Once the standard curves are drawn, the R^2^ results are obtained from the Corbett Research Thermal cycler machine software (Qiagen^®^). The R^2^ is based on the slope of the line; it is calculated by the formula:

$$Efficiency=10^{\left(-1|slope\right)}$$ (Eq 3)

PCR efficiency refers to the number of cycles required for DNA sequences to double under ideal conditions [28]. The integrity of the data fit to the theoretical line is described by the R^2^; this is a measure of the accuracy of the dilutions and precision of pipetting. R^2^ of 1.00 depicts a perfect assay [27].

After the standard curves are created dilutions of the reference DNA are included with each q-PCR reaction so that when the *eaeA* and *gadAB* standard curves are imported the results for the unknown samples are adjusted to obtain value/copies for the unknown samples, which in turn is used in calculating the ratio between *eaeA*: *gadAB*. The ratios between *eaeA* and *gadAB* were calculated using the following equation for all unknown samples:

$$Gene\;ratio=\frac{Calculated\;gadAb\;concentration}{Calculated\;eaeA\;concentration}$$ (Eq 4)

Where the gene concentrations are given as copies/3μl.

The LinRegPCR analysis program that was compared to the Corbett Research Thermal Cycler machine software works as follow:

The Raw data before baseline was corrected, exported from the Corbett Research Thermal cycler machine software, and imported into the LinRegPCR analysis program. Once analysed with the LinRegPCR program the results are provided as starting concentration (N0) based on the mean PCR efficiency of the amplicon. The advantage of using the N0 values is that the differences in PCR efficiency that can occur between your standard plasmids and the samples do not affect the result and that this method provides the lowest variation of q-PCR results [11]. The initial Eq 5 [22] indicated that the starting concentration of amplicon A (N0_A_) can be expressed relative to that of the reference amplicon (N0_B_) by direct division of these starting concentrations [11].


$$\frac{{N0}_{A}}{{N0}_{B}}$$ (Eq 5)

In this study, Eq 5 was adapted to calculate the ratios between *eaeA* and *gadAB*:

$$Value=\frac{{eaeA\;N0}_{Unknown}}{{eaeA\;N0}_{Reference}}$$ (Eq 6A)

$$Value=\frac{{gadAB\;N0}_{Unknown}}{{gadAB\;N0}_{Reference}}$$ (Eq 6B)

Where N0_Unknown_ is the *eaeA* gene 1 and *gadAB* gene 2, N0_Reference_ is the dilution of the reference DNA.

#### Statistical analysis

Statistical analysis was performed using the Graphpad Prism^®^ 7 and IBM SPSS statistics 23 software. The normality (Shapiro-Wilk test) and homogeneity of variances (Levine’s test) were tested to allow for further analysis for one-way ANOVA and Post-hoc tests. The non-parametric test was used to check for any contradictions to the parametric tests because there were less than 30 observations per sample. The Kruskal-Wallis tests was used to see if there are significant differences in the mean scores on the dependant variables across the groups. This test is an alternative to one-way ANOVA. The Mann-Witney U test was used to find out where these differences lie. This test is an alternative to the Post-hoc test.

## Results and discussion

### Obtaining ratio’s between *eaeA* and *gadAB* with absolute quantification

Seventy-four clinical *E*. *coli* strains were tested with the q-PCR targeting the *eaeA* and *gadAB* gene to determine the ratios the genes occur in “Table 2”. The *eaeA* gene was selected as a representative for the other *E*. *coli* pathotype genes because it is found on the chromosome of EHEC and EPEC. The acid-tolerance gene *gadAB* is found in all *E*. *coli* strains and was selected as the reference gene for all the experiments [29–31].

**Table 2:** Statistical analysis from linear regression between standard curves 1 to 4 for triplicates and 2 repeats, respectively.

Standard curve		Triplicate q-PCR analysis of each dilution				Standard curve repeats			
Nr	Description	Slope	R^2^	% EFFCY*	b^#^	Slope	R^2^	% EFFCY*	b^#^
1	Extracted DNA diluted in a buffer.	-3.4	0.99	97	20	-3.7	0.99	86	20
2	Extracted DNA diluted in extracted coliform DNA.	-3.7	0.99	86	23	-3.8	0.96	83	23
3	Cells diluted in coliform broth followed by DNA extraction from each dilution.	-3.8	0.98	83	20	-3.3	0.97	100	19
4	Cells diluted in water followed by DNA extraction from each dilution.	-4	0.97	79	20	-4.4	0.93	69	20

*PCR efficiency

^#^y-intercept.

**Fig 2:**
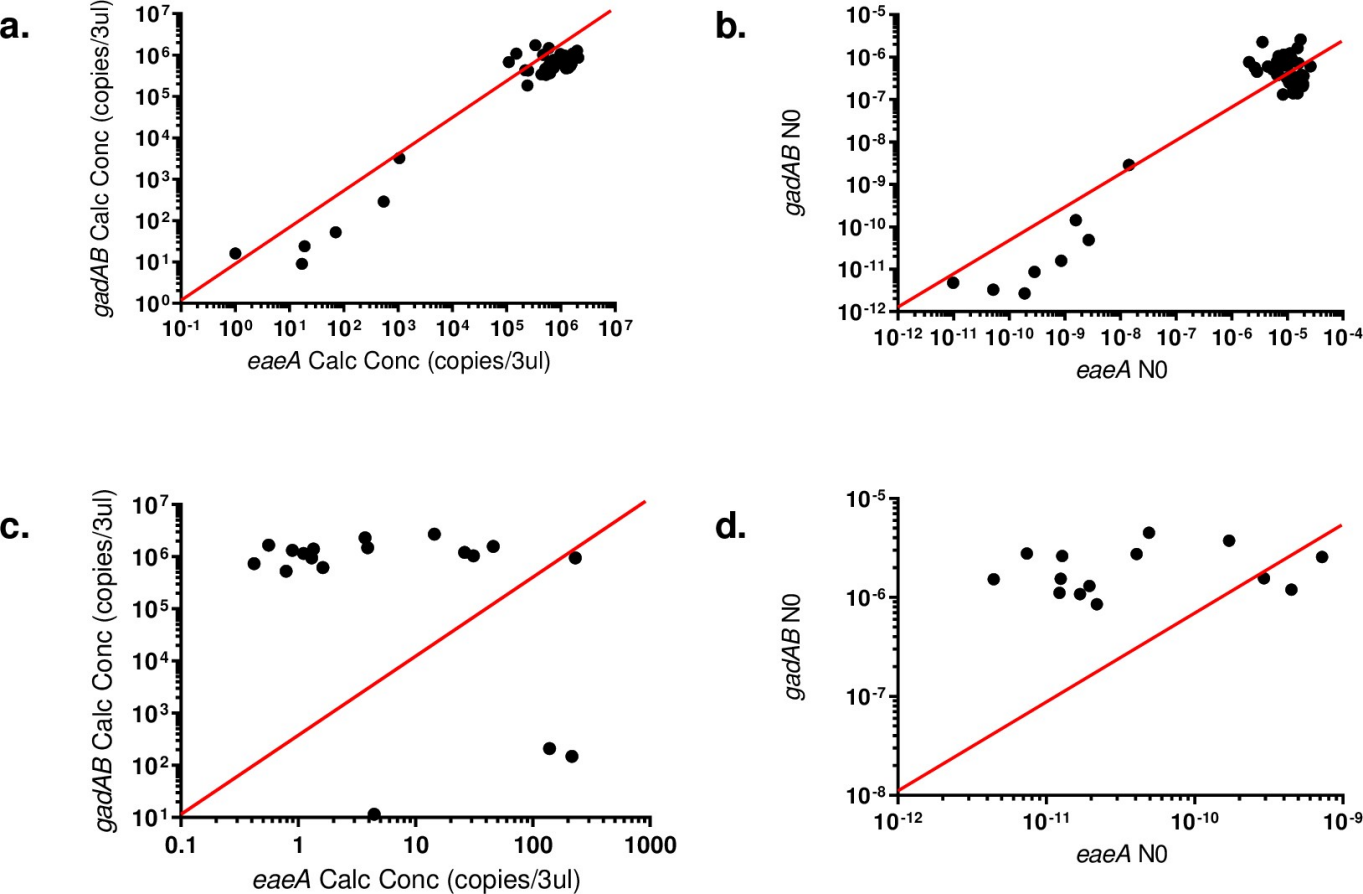
Graphs depicting the *gadAB vs eaeA* copies for the 2005–2006 clinical strains (a) and 2008–2009 clinical strains (c) drawn with the Corbett Research Thermal cycler software and *gadAB* vs *eaeA* N0 calculated with the LinRegPCR software for the 2005–2006 clinical strains (b) and 2008–2009 clinical strains (c). The red reference line in each graph indicates in each graph is when x = y.

The *eaeA* and *gadAB* standard curves were constructed using diluted extracted DNA with the standard curves constructed by the Corbett Research Thermal cycler software “Standard curve 1, Fig 4A”. The R^2^ for the two curves was 0.999 (*eaeA*) and 0.997 (*gadAB*) with a slope of -3.32 (*eaeA*) and -3.6 (*gadAB*) and a y-intercept of 30.25 (*eaeA*) and 36.29 (*gadAB*). The gene copies obtained for the *eaeA* and *gadAB* for the two groups of strains is graphically shown in “Fig 2A and 2C”. Using Eq 4 provided the *eaeA*: *gadAB* ratios were calculated for each of the 74 strains and ranged between 0.37 to 20.51 for the 2005–2006 clinical strains and 33504 to 2967937 for the 2008–2009 clinical strains.

The gene copy numbers and *eaeA*: *gadAB* ratios were also calculated with the LinRegPCR analysis program [22] to see if similar results could be obtained. The gene copies calculated for the *eaeA* and *gadAB* genes are shown in “Fig 2B and 2D” for the two groups of clinical isolates and visually shows comparable results with the ratio calculated with the Corbett Research Thermal cycler software. When calculating the the *eaeA*: *gadAB* ratios it ranged between 0.01–0.64 for the 2005–2006 strains and 2671–345654 for the 2008–2009 strains.

**Fig 3:**
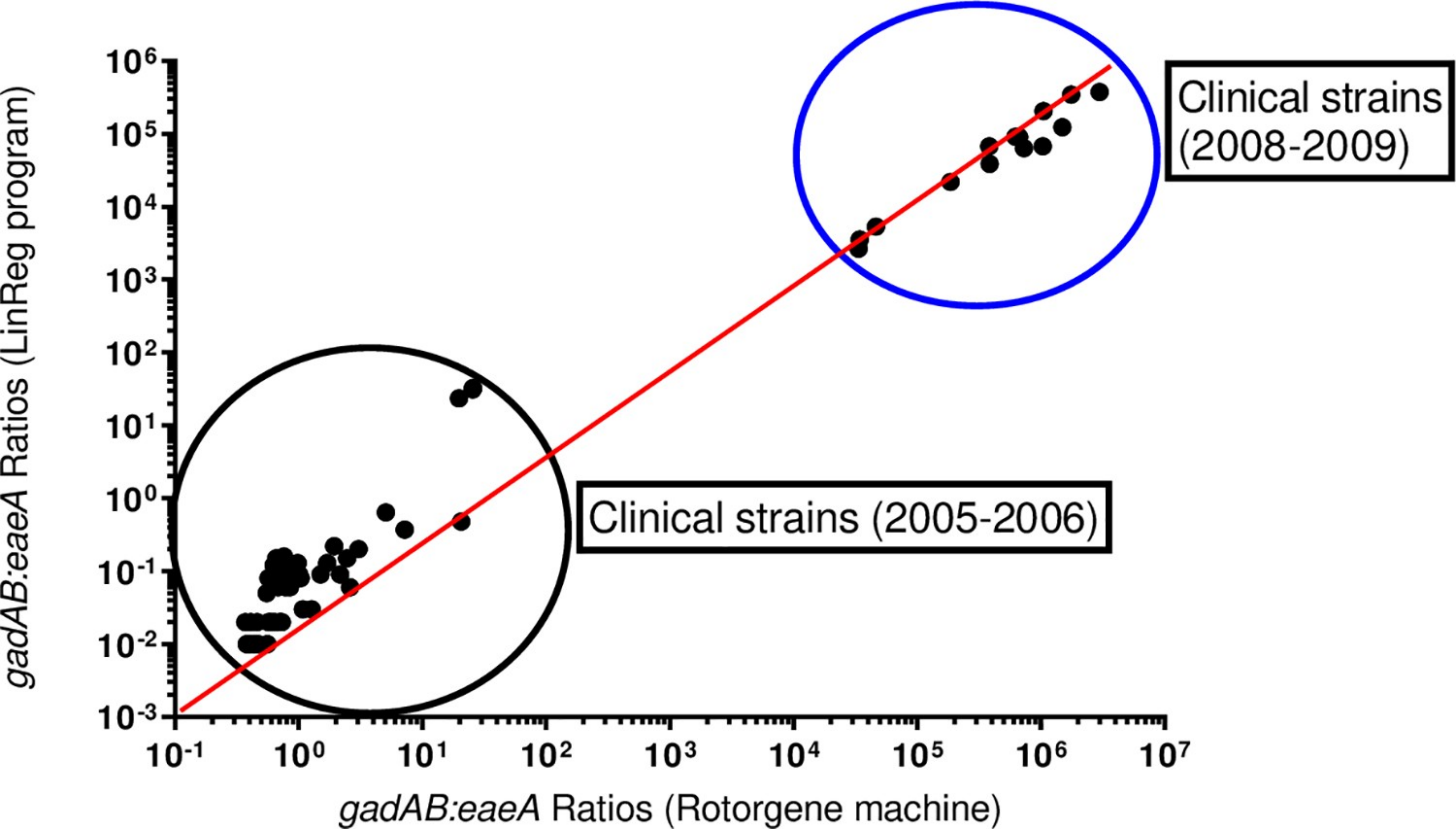
Graphs showing a comparison of the gene ratios as determined with Corbett Research Thermal cycler software versus the LinRegPCR program for combination *gadAB* vs *eaeA* for clinical strains (2005–2006) and clinical strains (2008–2009). The linear line indicated in each graph in red is a reference line where x = y.

The gene ratio results from the two programs were combined to create a scatter plot as shown in “Fig 3”. Although there is a 1 log_10_ difference between the ratios calculated with each program, with the Corbett Research Thermal Cycler ratios being higher, they each still created the same clusters of the 2005–2006 and 2008–2009 clinical strains. Since these clusters differ between two groups of *E*. *coli* it could be used for an early grouping of specific strains or isolates. In this case, the difference could have been between the strains being typical or atypical EPEC. All the 2008–2009 clinical strains were confirmed as aEPEC [32], however, for the 2005–2006 clinical strains the strains were only confirmed as EPEC [33].

The LinRegPCR was not able to calculate the ratios for seven (9.5%) of the isolates with the program assigning the samples as “noisy”. According to the manual, the reason for noisy samples is poor data quality that is excluded from the calculation of the mean efficiency [34]. Based on the data obtained with the Corbett Research Thermal Cycler ratios six of the samples would have grouped with the 2005–2006 clinical isolates and the last one with the 2008–2009 isolates.

### Comparison of various standard curves for the *eaeA* gene

The influence of both bacterial cell’s concentration in the samples before DNA extraction, as well as the influence of other bacteria on the DNA extraction, needs to be studied as this will influence the q-PCR, the standard curve constructed and downstream processes. The data for the q-PCR reproducibility (triplicate q-PCR analysis of each dilution) “Table 2” shows that factors such as DNA extraction of diluted cells, free DNA and other bacteria cells do influence the DNA extraction and thus the PCR efficiency that drops from 97% for standard curve 1 to 79% for standard curve 4. This in turn influences the goodness of fit of the standard curves and the slopes and y-intercepts. Similar trends are seen when comparing the data for the two repeats of the standard curves created where the mean PCR efficiency is lower than the duplicate repeats ranging between 69–100% efficiency. Standard curve 3 had a 100% PCR efficiency that can be explained by the presence of background cells that assisted with the DNA extraction efficiency of the lower *E*. *coli* dilutions. Other authors have reported on the impact of carrier nucleic acids on recovering low levels of pathogens from samples [35]. The addition of the Coliform cells, similar to what would be found in the IDEXX Colilert^®^ Quanti-Tray^®^ may indirectly assist with the DNA extraction method.

Statistical analysis of the data in “Table 3” shows that when the standard curves are compared, there was no significant difference between standard curves prepared with diluted DNA or with cells diluted before the DNA is extracted (P = 0.435) “Table 3”. However, there were significant differences when background DNA was included in the diluent or Coliform cells added to the diluent to dilute cells before the DNA is extracted (P < 0.001). This further support the observation that carrier DNA does influence the recovery of the *E*. *coli* DNA and thus the standard curves.

**Table 3:** Statistical analysis between the four standard curves.

C_q_	Coefficient of variance	P>[z]	95% conf. interval
Comparison between DNA extracted then diluted and cells diluted then extracted.	-0.429	0.435**	-1.509 to 0.649
Comparison of std. curves with and without background DNA.	3.538	<0.001*	2.459 to 4.617
Comparison between both methods described above.	-2.771	<0.001*	-4.298 to 1.245

P ≥ 0.05 non-statistically different**; P < 0.05 statistically different*.

**Fig 4:**
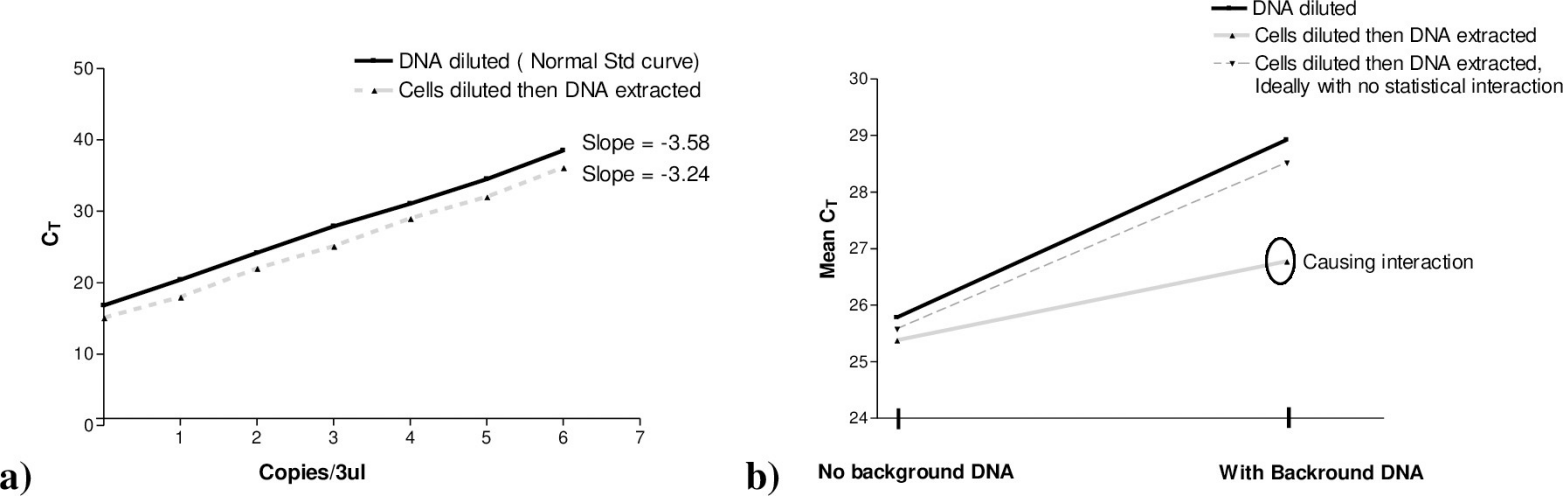
a) Reported normal standard curve and cells diluted then DNA extracted standard curve from literature; b) Statistical interactions between the four standard curves.

The statistical interaction (Mean C_q_ values) between the four standard curves was analysed and is presented graphically in “Fig 4B”. The statistical interaction indicates that extracted individual DNA dilutions with background DNA cause interaction i.e., there are differences between the standard curves “Grey line in Fig 4B”, therefore, it does not lie parallel to the diluted DNA “Fig 4A”. Statistical comparisons further indicate the mean C_q_ values were better for extracted individual DNA dilutions with background DNA than without background DNA (between C_q_ 25–28) “Fig 4B”, further supporting what was observed earlier.

The results support other authors that recommended the inclusion of carrier DNA [35, 36] and extracting the DNA from diluted cells also suggested extracting individual dilutions to fully characterise the variability in the DNA extraction [37]. The influence of other types of inhibitors not removed with the DNA extraction on the PCR reaction was not considered, but it has been shown that the addition of α-casein can assist with removing inhibitors from human and environmental samples [38, 39].

## Conclusion

Both the Corbett Research Thermal Cycler software and LinRegPCR software gave similar gene ratios although there was a log_10_ difference in the values. Despite this, both the programs were able to group the same clinical strains together and the use of gene ratios in mixed samples could be a fast robust method to test for strain relatedness between samples. It is important that the correct method for constructing standard curves for an environmental sample is chosen because the bacterial cell concentration and presence of bacteria do influence the DNA extraction and subsequent PCR efficiency. Overall, the results show that using standard curve three is the best option when using samples enumerated with the IDEXX Colilert^®^ Quanti-Tray^®^ system.
